# Transcallosal and Pericallosal Courses of the Anterior Cerebral Artery

**DOI:** 10.3390/medicina58101365

**Published:** 2022-09-28

**Authors:** Dragoş Ionuţ Mincă, Mugurel Constantin Rusu, Petrinel Mugurel Rădoi, Sorin Hostiuc, Corneliu Toader

**Affiliations:** 1 Division of Anatomy, Department 1, Faculty of Dental Medicine, “Carol Davila” University of Medicine and Pharmacy, RO-020021 Bucharest, Romania; 2 Division of Neurosurgery, Department 6—Clinical Neurosciences, Faculty of Medicine, “Carol Davila” University of Medicine and Pharmacy, RO-020021 Bucharest, Romania; 3 Clinic of Neurosurgery, “Dr. Bagdasar-Arseni” Emergency Clinical Hospital, RO-041915 Bucharest, Romania; 4 Department of Legal Medicine and Bioethics, Faculty of Dental Medicine, “Carol Davila” University of Medicine and Pharmacy, RO-020021 Bucharest, Romania

**Keywords:** internal carotid artery, anterior cerebral artery, corpus callosum, neurosurgery, interventional radiology

## Abstract

(1) Background: The anterior cerebral artery (ACA) has a precommunicating A1 segment, followed by a postcommunicating A2 segment. Anatomically, after it sends off from the callosomarginal artery (CMA), it continues as the pericallosal artery (PCalA). A detailed pattern of the anatomical variations of the PCalA are needed for practical reasons. (2) Methods: There were 45 retrospectively documented Computed Tomography Angiograms of 32 males and 13 females. (3) Results: In 90 sides, eleven different types of PCalA were documented: type 1: normal origin, above the genu of the corpus callosum (CC) (51.11%); type 2: low origin, below the rostrum of the CC (8.88%); type 3: late origin, above the body of the CC (3.33%); type 4, initial transcallosal course (3.33%); type 5, duplicated PCalA (1.11%); type 6, azygos PCalA (2.22%); type 7, absent PCalA (CMA type of ACA) (7.78%); type 8: CMA continued as PCalA (5.56%); type 9: PCalA continued as the cingular branch (1.11%); type 10: PCalA type of ACA, absent CMA (14.44%); type 11: triple PCalA, with an added median artery of the CC (1.11%). Different types of CMA were also documented: type 0, absent CMA (17.78%); type 1, CMA with frontoparietal distribution (45.56%); type 2, CMA with parietal distribution (22.22%); type 3, low origin of CMA, either from A1, or from A2 (8.88%); type 4, CMA continued as PCalA (5.56%). Ipsilateral combinations of PCalA and CMA types were classified as types A-P. In 33/45 cases (73.3%), the bilateral asymmetry of the combined anatomical patterns of PCalA and CMA was documented. Additional rare variations were found: (a) huge fenestration of A2; (b) bihemispheric ACAs (6/45 cases); (c) twisted arteries within the interhemispheric fissure. (4) Conclusions: The PCalA and CMA are anatomically diverse and unpredictable. Therefore, they should be documented on a case-by-case basis before surgical or endovascular approaches.

## 1. Introduction

The internal carotid artery bifurcates into the anterior (ACA) and middle (MCA) cerebral arteries [1]. The ACA begins at the medial end of the trunk of the lateral cerebral fissure [2]. From its origin, the ACA runs posteriorly above the corpus callosum (CC), vascularizing most of the medial aspect of the cerebral hemisphere. The artery irrigates the medial regions of the frontal and parietal cortex, the CC and the falx cerebri [3]. The ACAs are united by the anterior communicating artery (AComA) which builds the anterior side of the circle of Willis.

There are several classification schemes for the segments of the ACA [4]. A simple classification divides the ACA into three portions. The A1 segment is the precommunicating segment of the ACA. The A2 segment holds from the junction with the AComA to the origin of the callosomarginal artery (CMA), being the vertical or postcommunicating segment. The course of the CMA corresponds to the cingulate sulcus above the cingulate gyrus, on the medial aspect of the hemisphere [2,5]. The A3 segment is distal to the origin of the CMA; this segment is also known as the pericallosal artery (PCalA) [1,2,6] as it courses into the pericallosal sulcus [7]. The cingulate gyrus separates the CMA and PCalA. The term “pericallosal artery” is often used inaccurately for the A2 segment [8,9,10,11]. 

An azygos ACA is an uncommon anomaly in which the A1 segments of both ACAs unite into a single A2 segment with bilateral distribution, being prone to the formation of aneurysms [12]. Differently, a bihemispheric ACA (asymmetric ACA) is defined by the combination of one dominant A2 segment supplying most of the cortical territory of both ACAs [13]. An azygos PCalA leaves an A2 segment to further divide into the left and right PCalAs [14]. Thus, an azygos PCalA is a bilaterally distributed PCalA [11,15], being mandatorily flanked by initial trunks of cortical branches, such as the CMAs. 

The increasing use of the operating microscope for deep approaches has created a need for a better understanding of the microsurgical anatomy of the ACA [10]. We hypothesized that the topographical and morphological possibilities of the distal ACA could be diverse, and thus aimed at studying the patterns of ACA branching on computed tomography angiograms (CTAs).

## 2. Materials and Methods

A randomly selected retrospective lot of 45 CTAs were studied. Of these, 32 were in male cases and 13 in female cases. Inclusion criteria were the age of the subjects (>18 years), adequate quality of the CTAs, and no pathologic processes distorting the vascular anatomy in the territory of the ACA. Exclusion criteria were pathological processes distorting the vascular anatomy, and degraded or incomplete CTAs. 

The research was conducted following principles of the World Medical Association Code of Ethics (Declaration of Helsinki). All subjects gave their informed consent for inclusion before they participated in the study. The responsible authorities (affiliation 3) approved the study (approval no. 2093/1 March 2022).

CT scans were performed with a 32-slice scanner (Siemens Multislice Perspective Scanner), with a 0.6 mm collimation and a reconstruction of 0.75 mm thickness with 50% overlap for a multiplanar maximum intensity projection and three-dimensional volume rendering technique, as described previously [16]. The cases were documented using Horos for iOS (Horos Project).

## 3. Results

Eleven anatomical types of PCalA were documented: type 1: normal origin, above the genu of the corpus callosum (CC); type 2: low origin, below the rostrum of the CC (either 2a, from A1 ACA, or 2b, from the initial segment of A2 ACA); type 3: late origin, above the body of the CC; type 4: initial transcallosal course, further continued as pericallosal; type 5: duplicated PCalA; type 6: azygos PCalA, single initial trunk, further divided in two branches with pericallosal course; type 7: absent PCalA, CMA type of ACA; type 8: CMA continued as PCalA; type 9: PCalA continued as the cingular branch; type 10: PCalA type of ACA, absent CMA. The triple PCalA variant was recorded as type 11.

Several variants of CMA were found: type “0”: absent CMA; type “1”: CMA with frontoparietal distribution; type “2”: CMA with parietal distribution; type “3”: low origin of the CMA, either from the A1 ACA (subtype 3a), or from the initial part of the A2 ACA (subtype 3b). When the CMA continued as PCalA, the CMA was recorded as type “4”. 

**Figure 1:**
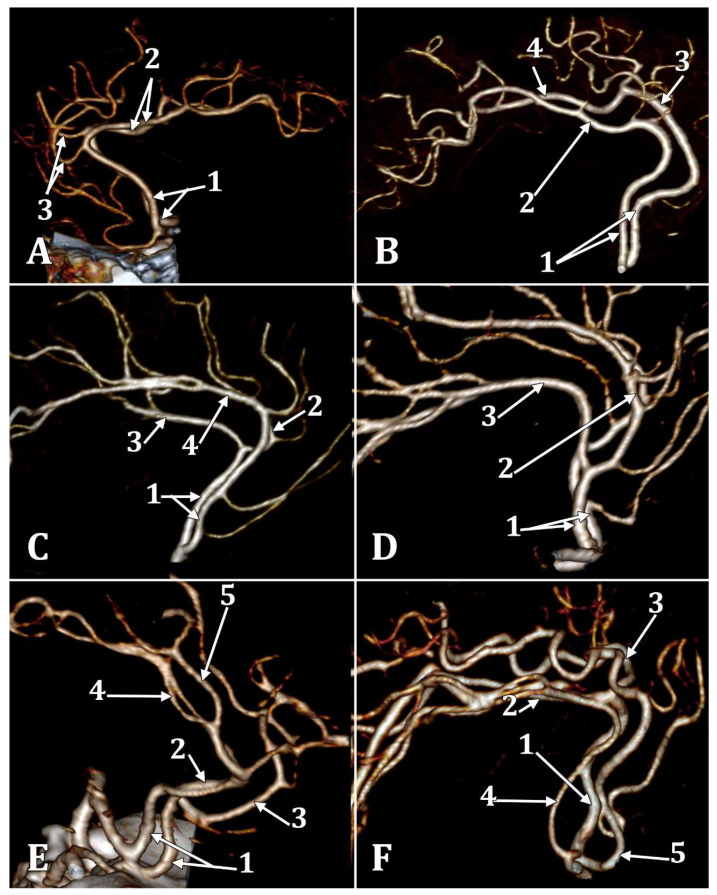
Three-dimensional volume renderings of different types of different branching types of the anterior cerebral arteries (ACAs) into the pericallosal (PCalA) and callosomarginal (CMA) arteries. (**A**) Left antero-lateral view: 1 A2 segments of the ACAs; 2. type 1 PCalAs; 3. type 1 CMAs. (**B**) Right lateral view: 1. A2 segments of the ACAs; 2. type 10 right PCalA (PCalA type of ACA); 3. type 4 left CMA, at the level of the cingulate sulcus; 4. type 8 left PCalA. (**C**) Right lateral view: 1. A2 segments of the ACAs; 2. left type 1 CMA; 3. left type 1 PCalA; 4. right type 1 CMA, type 7 (absent) right PCalA. (**D**) Right lateral view: 1. A2 segments of the ACAs; 2. type 3b of the right CMA; 3. type 2b of the right PCalA. (**E**) Right antero-lateral view: 1. A2 segments of the ACAs; 2. type 2a of the left PCalA; 3. type 3a of the left CMA; 4. type 3 of the right PCalA; 5. type 2 right CMA. (**F**) Right lateral view: 1. A2 segment of the left ACA; 2. left type 1 PCalA; 3. left type 1 CMA; 4. type 2a right PCalA; 5. type 3a right CMA.

**Figure 2:**
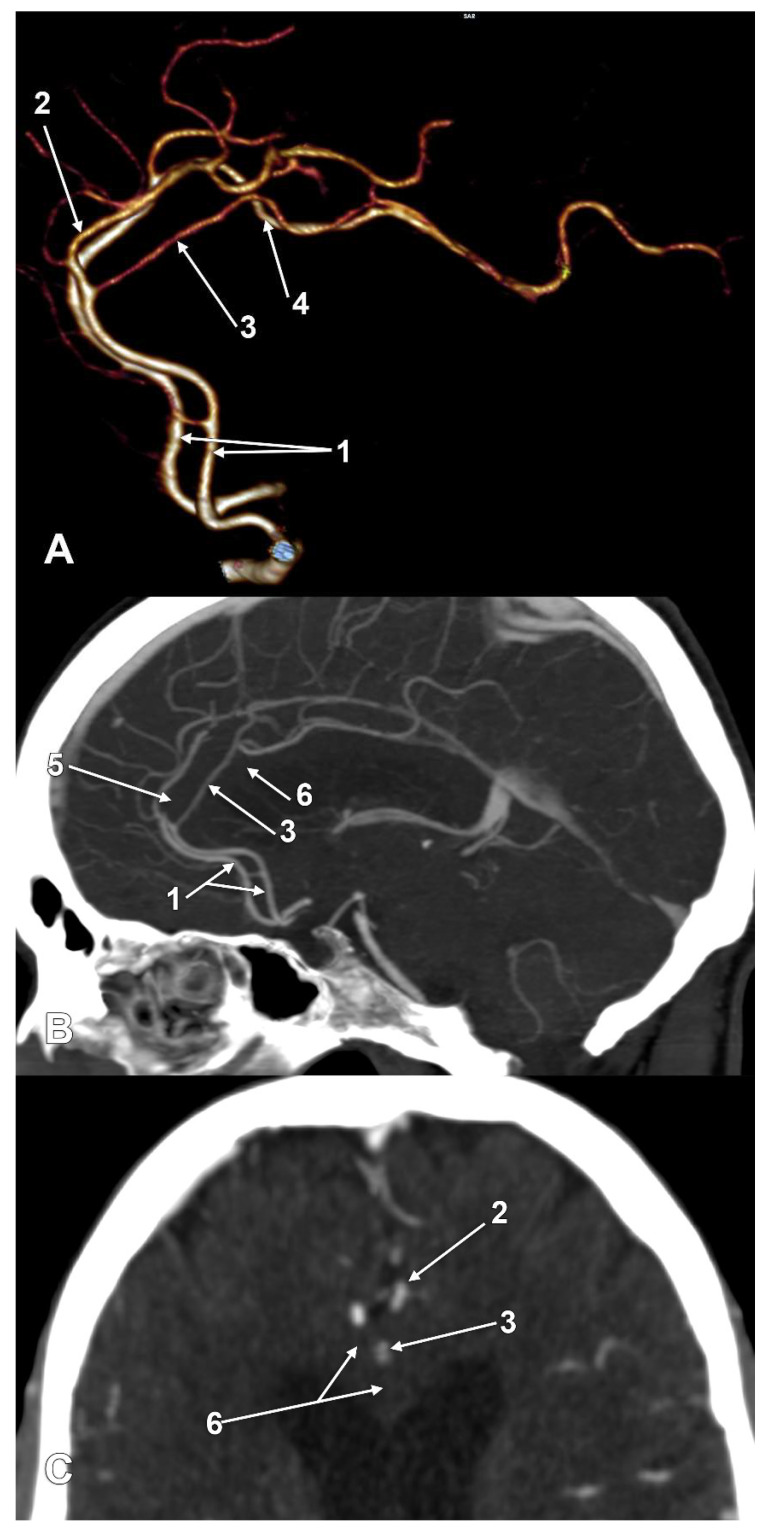
Type 4, pericallosal artery coursing through the corpus callosum. (**A**) Three-dimensional volume rendering, left lateral view; (**B**) Mediosagittal slice through the corpus callosum. (**C**) Axial slice through the corpus callosum: 1. anterior cerebral arteries; 2. left callosomarginal artery; 3. transcallosal course of the left pericallosal artery; 4. right pericallosal artery; 5. genu of corpus callosum; 6. body of corpus callosum.

**Figure 3:**
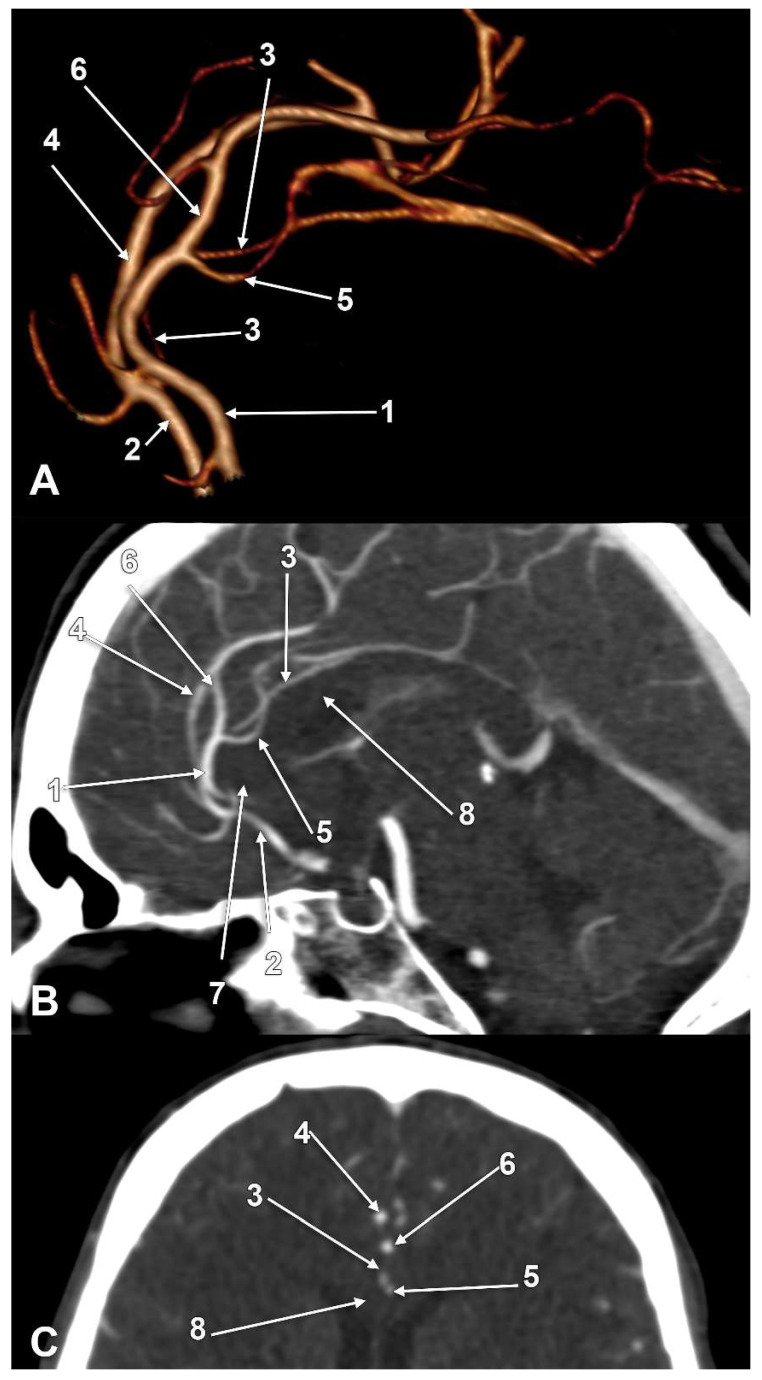
Type 4 pericallosal arteries coursing through the corpus callosum; (**A**) Three-dimensional volume rendering, left lateral view; (**B**) Mediosagittal slice through the corpus callosum; (**C**) Coronal slice through the corpus callosum: 1. left anterior cerebral artery; 2. right anterior cerebral artery; 3. right pericallosal artery; 4. right callosomarginal artery; 5. transcallosal course of the left pericallosal artery; 6. left callosomarginal artery; 7. rostrum of corpus callosum; 8. body of corpus callosum.

**Figure 4:**
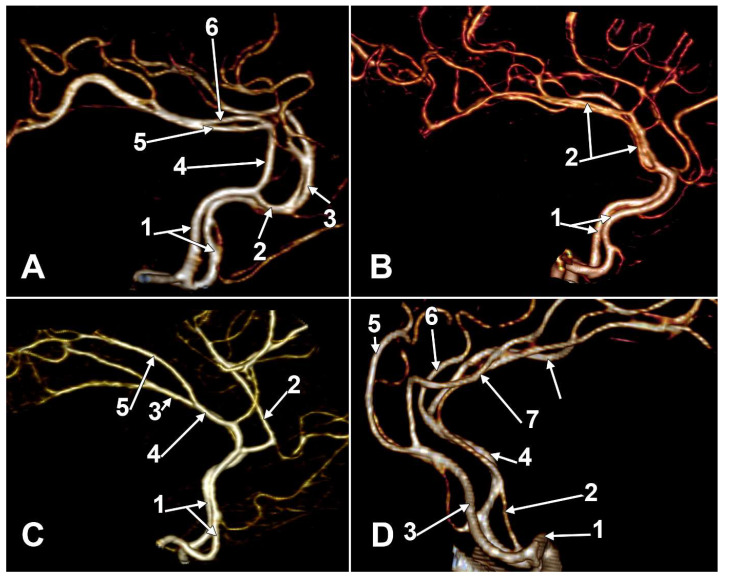
Three-dimensional volume renderings of different types of different branching types of the anterior cerebral arteries (ACAs) into the pericallosal (PCalA) and callosomarginal (CMA) arteries. (**A**) Right lateral view: 1. A2 segments of the ACAs; 2. type 1 right CMA; 3. type 1 left CMA; 4. type 6 type 6 PCalA (azygos PCalA); 5. right PCalA; 6. left PCalA. (**B**) Right lateral view: 1. A2 segments of the ACAs; 2. type 5 (duplicated) right PCalA. (**C**) Right lateral view: 1. A2 segments of the ACAs; 2. left CMA; 3. left PCalA; 4. type 9 right PCalA; 5. right cingular branch. (**D**) Left lateral view, type 11—triple PCalA: 1. A1 segment of bihemispheric right ACA; 2. median callosal artery; 3. left A2 segment; 4. right bihemispheric A2 segment; 5. left CMA; 6. right CMA; 7. left PCalA; 8. right PCalA.

The incidences of the PCalA types, as defined in the 90 sides that were investigated, resulted in a decreasing order, as follows: PCalA type 1 (Figure 1A,C,F) was found in 46/90 sides (51.11%), type 10 (Figure 1B) was found in 13/90 sides (14.44%), type 7 (Figure 1C) was found in 7/90 sides (7.78%), type 2b (Figure 1D) was found in 6/90 sides (6.67%), type 8 (Figure 1B) was found in 5/90 sides (5.56%), types 3 (Figure 1E) and 4 (transcallosal course of the PCalA) (Figure 2 and Figure 3, Video S1) were found each in 3/90 sides (3.33%), types 2a (Figure 1E,F) and also 6 (Figure 4A), were found in 2/90 sides (2.22%), and types 5 (Figure 4B), 9 (Figure 4C), and 11 (triple PCalA) (Figure 4D) were each found in 1/90 sides (1.11%) (Table 1).

The CMA types were determined with the following incidences, in decreasing order: type 1 (Figure 1A,C,F)—41/90 sides (45.56%), type 2 (Figure 1E)—20/90 sides (22.22%), type 0 (absent CMA)—16/90 sides (17.78%), types 3b (Figure 1D) and also type 4 (Figure 1B), in 5/90 sides (5.56%), and type 3a (Figure 1E,F) in 3/90 sides (3.33%) (Table 1). 

On each side, right and left, were recorded several combinations of PCalA and CMA types (Table 1). The ipsilateral combinations of the PCalA and CMA types were classified as types A-P (Table 2).

In just 8/32 male cases, and in 4/13 female cases, the combinations of PCalA and CMA types were bilaterally symmetrical. Therefore, in 33/45 cases (73.3%), 24 male and 9 female, the bilateral asymmetry of the combined anatomical patterns of PCalA and CMA were documented. The detailed gender distribution of the bilateral combinations of the types A-P is presented in Table 3.

**Figure 5:**
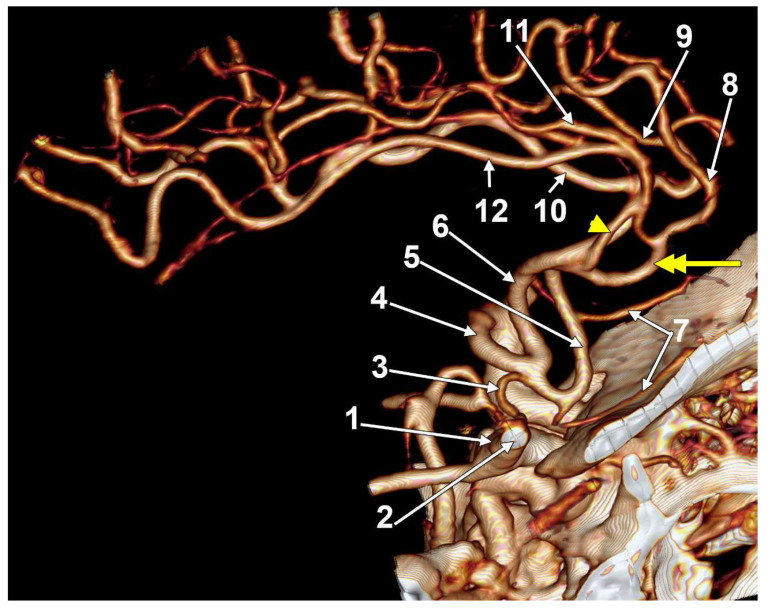
Three-dimensional volume rendering, right lateral view of the anterior cerebral arteries (ACAs): 1. right internal carotid artery; 2. right middle cerebral artery; 3. hypoplastic A1 segment of the right ACA; 4. A1 segment of the bihemispheric left ACA; 5. right A2 segment; 6. left A2 segment; 7. bilateral orbitofrontal branches; 8. right callosomarginal artery; 9. left callosomarginal artery; 10. left pericallosal artery; 11. right cingular branch; 12. right pericallosal artery. A huge fenestration of the right A2 segment consists of a postero-lateral arm (arrowhead) and an antero-medial one (double-headed arrow). The A2 segments are twisted in the interhemispheric fissure.

In six cases, 5 males and 1 female, bihemispheric ACAs were found. The dominant ACA was on the right side in three cases, and on the left side in the other three cases. In two cases with right bihemispheric ACA, additional variants were found: (a) a median artery of the CC added to the bilateral PCalAs (type 11 of PCalA, triple PCalA, Figure 4D), and (b) a huge fenestration of the A2 segment (Figure 5).

Twisted arterial trunks were found in the anterior interhemispheric fissure, in a combined type F female case (Figure 1E)

In a male case with bilateral PCalA/CMA type A combinations of types, a peculiar arterial morphology was found (Figure 5), consisting of a left bihemispheric ACA connecting a right hypoplastic A1 segment. The left ACA supplied both A2 segments, right and left, that further coursed twisted in the interhemispheric fissure. On the right A2 segment, a large fenestration was found, consisting of postero-lateral and antero-medial arms. From the antero-medial arm of that fenestration left the right CMA. From the distally reunited arms of that fenestration resulted a trunk that was further dividing into the right cingular artery and right PCalA.

## 4. Discussion

Rare anatomical variants in the territory of the ACA were found during the present study, such as the transcallosal course of the PCalA, duplicated PCalA, azygos PCalA, triple PCalA, CMA continuing as PCalA, or PCalA continuing as the cingular branch (groove gliding of PCalA), huge A2 fenestration, and twisted arteries of the anterior interhemispheric fissure. Such arterial variants are surgically important as they lie below the caudal free margin of the falx cerebri and are exposed in microsurgical approaches to the sellar and chiasmatic regions, pineal gland, or the third and lateral ventricles [17]. Interventional neuroradiologists should document each case without assuming a certain general pattern. 

The CMA is the second-largest distal branch of the ACA, after the PCalA [5]. According to different authors, the CMA is absent in 15%–40% of cerebral hemispheres [5,11,18]. We found the absence of the CMA in 17.7% of cases. Moreover, our findings demonstrated that, if present, the CMA has a certain topographic variability. The anatomy of the CMA was also documented in fetuses [19]. The absence of the CMA was found in 36.06% of 452 fetal brains, being also found that the site of origin of the CMA has a possible proximal-to-distal variation [19]. The possible absence of the CMA was brought forward as an argument for using the term PCalA for the A2 postcommunicating segment of the ACA [20]. 

A subfalcine hernia could complicate with the compression of the ACA branches, especially the PCalA, leading to a cerebral infarct. However, this would not be a common consequence because, as we demonstrated here, the PCalA is subjected to different variations that include its absence. The anatomical variability of the PCalA also involve heterogeneous possible symptoms of specific vascular compression.

Inconsistencies regarding the anatomical definition of the PCalA (see Introduction) consequently induce variable definitions of an absent PCalA. When the PCalA is erroneously regarded as the A2 segment, an absent PCalA is an absent A2 segment, thus the contralateral ACA is an azygos, or bihemispheric ACA. However, no matter the anatomical distinction, if an endovascular approach for an aneurysm of the distal PCalA is intended, the contralateral ICA would be used to gain access. 

An azygos PCalA, which was classified here as a type 6 PCalA, was previously found in 3/112 brains (2.7%) [14] and in 1/38 brains (2.63%) [18]. We found it here in 2.2% of cases, and are thus confirming that it is a rarely occurring anatomical variant. Although being rare, it is important to identify it as a unique callosal source, especially when it accommodates aneurysms, because damaging it could lead to an isolated CC infarction [21,22].

It was found here in just a single case (1.11%), with a triple PCalA (type 11). In that case, a median artery of the CC left the AComA. In more than 900 patients, the median artery of the CC had an incidence of 3.0% [23]. To indicate the median artery of the CC, different terms were used, such as “median callosal artery”, “third A2” segment, “accessory ACA”, “*arteria cerebralis anterior media*”, or triple/triplicated ACA [24,25]. 

The descriptions of the azygos PCalA and the median artery of the CC could fit [11]. However, when an azygos PCalA leaves an A2 segment, the opposite A2 segment sends off only the CMA, while the left and right PCalAs have a late origin, above the CC [14]. When present, the median artery of the CC arises from the AComA [19,24,25,26] and not from the A2 segment. This helps in distinguishing it from an azygos PCalA leaving one of the ACAs. This may not be exclusive, since an azygos PCalA was found leaving the AComA, and not an A2 segment [18]. The median artery of the CC represents a persisting fetal pattern [11,19].

A triple PCalA was found in this study. It consisted of the left and right PCalAs and an added median artery of the CC. Such a rare anatomic variant should be detected before an endovascular route approach, in order to understand the correct pathway towards an eventual aneurysm of the distal PCalA. 

PCalAs with transcallosal courses were found in two cases, unilateral and bilateral. To our knowledge, such peculiar variants have not been previously reported. Where the PCalAs are found to be distorting the anatomy of the CC, this was the only case reported recently by Zytkowski et al. (2022), who found a peculiar bilateral variant of the PCalA by dissection: when these arteries reached above the corpus of the CC they looped inferiorly to determine a deep hollow within the CC, thus decreasing the thickness of the CC from 6.8 to 1.5 mm [6]. As Zytkowski concluded, arterial anomalies could alter the brain tissue [6] and therefore could determine apparently unexplained symptoms. 

Motor dysfunction within the lower limb might be provoked by stenosis of the PCalA [6]. Stenosis of the supracallosal segment of the PCalA could also determine the callosal type of alien hand syndrome (intermanual conflict, the non-dominant hand being usually affected) [22].

Arteries are normally straight conduits, but tortuous blood vessels are a common angiographic finding, being increasingly detected with the advance of imaging technology [27]. Twisted atherosclerotic vessels lead to sudden increases in blood flow and resulting mechanical impact on the adjacent brain areas [28]. Moreover, during neurosurgical or endovascular approaches, twisted vessels within the anterior communicating system, such as were found in this study, could be misidentified on the wrong side.

Fenestration of the ACA is a rare finding [29]. Fenestrations of the ACA usually involve the A1 segment [29,30,31,32]. Bilateral A1 fenestrations were also reported [32,33], as well as the double fenestration of the A1 segment [34] and the fenestration of a median artery of the CC [35]. Fenestrated azygos ACAs were also found [36,37]. In 2/891 cases, small slit-like fenestrations of the A2 segment of the ACA were found [23]. A2 fenestrations were found at the level of the AComA, thus in a low location on the A2 segment [38]. To our knowledge, a high and large fenestration of the A2 segment, such as was found in the present study, has not been previously reported. This variant, although extremely rare, should be considered when multiple and, occasionally, twisted arterial trunks are found intraoperatively within the interhemispheric fissure. 

## 5. Conclusions

Seemingly, the textbook arterial morphology of the distal ACA branches, as is the type 1 PCalA we defined, could be encountered in just half of cases. The true heterogeneous anatomical possibilities, as well as combinations of individual PCalA and CMA variants, indicate that the anatomy of the ACA is quite diverse and unpredictable. The combinations of types are rather bilaterally asymmetrical. Therefore, the arterial anatomy in the ACA territory should be documented case-by-case instead of assuming it having a certain anatomical pattern.

## Figures and Tables

**Table 1:** Types of pericallosal (PCalA) and callosomarginal (CMA) arteries that were found in 90 sides.

PCalA Types	PCalA Types Incidence	CMA Types	CMA Types Incidence
“1”: normal origin, above the genu of the corpus callosum	51.11%	type 0: absent CMA	17.78%
“2a”: low origin, below the rostrum of the corpus callosum, from the A1 ACA	2.22%	type 1: CMA with frontoparietal distribution	45.56%
“2b”: low origin, below the rostrum of the corpus callosum, from the proximal A2 ACA	6.67%	type 2: CMA with parietal distribution	22.22%
“3”: late origin, above the body of the corpus callosum	3.33%	type 3a: CMA from A1 ACA	3.33%
“4”: initial transcallosal course, further continued as pericallosal	3.33%	type 3b: CMA from the proximal A2 ACA	5.56%
“5”: duplicated PCalA	1.11%	type 4: CMA continued as PCalA	5.56%
“6”: azygos PCalA	2.22%		
“7”: absent PCalA	7.78%		
“8”: CMA continued as PCalA (type 4 CMA)	5.56%		
“9”: PCalA continued as the cingular branch	1.11%		
“10”: PCalA type of ACA	14.44%		
“11”: triple PCalA	1.11%		

**Table 2:** Gender distribution of ipsilateral combinations of pericallosal (PCalA) and callosomarginal (CMA) arteries types. Types of combinations are defined and range from A to P. M: male; F: female.

Combinations of Types	PCalA Type	CMA Type	RIGHT SIDE Count, Gender	LEFT SIDE Count, Gender
A	1	1	12M 3F	12M 3F
B	1	2	7M 2F	5M 2F
C	2a	3a	1M	1M
D	2b	3a	1M	0
E	2b	3b	2F	1M 2F
F	3	2	1M 1F	1M
G	4	1	1F	1M 1F
H	5	0	1F	0
I	6	0	0	1M
J	6	1	1M	0
K	7	0	1M	0
L	7	1	2M 1F	3M
M	8	4	2M 1F	1M 1F
N	9	2	1M	0
O	10	0	3M 1F	6M 3F
P	11	1	0	1F

**Table 3:** Gender and lot distribution of bilateral combinations of pericallosal-callosomarginal types.

Right/Left Combinations of Types	Male Cases	Female Cases	Total
A/A	5	1	6
A/A	6	1	7
A/C	1	0	1
A/E	0	1	1
A/F	1	0	1
A/L	1	1	2
A/M	0	1	1
A/N	1	0	1
A/O	4	0	4
B/B	1	0	1
B/F	1	0	1
B/H	0	1	1
B/L	3	0	3
B/O	0	1	1
B/P	0	1	1
C/D	1	0	1
E/E	0	1	1
E/F	0	1	1
E/M	1	0	1
G/G	0	1	1
G/M	1	0	1
J/L	1	0	1
K/I	1	0	1
O/O	2	1	3
O/M	1	1	2

## Supplementary Materials

The following supporting information can be downloaded at: https://www.mdpi.com/article/10.3390/medicina58101365/s1, Video S1: Transcallosal arteries.
